# Young children are the main victims of fast food induced obesity in Brazil

**DOI:** 10.1371/journal.pone.0224140

**Published:** 2019-10-22

**Authors:** Paul-Georges Reuter, Lucas Afonso Barbosa Saraiva, Lisa Weisslinger, Carla De Stefano, Frédéric Adnet, Frédéric Lapostolle

**Affiliations:** SAMU 93—UF Recherche-Enseignement-Qualité, Université Paris, Sorbonne Cité, Inserm U942, Hôpital Avicenne, AP-HP, Bobigny, France; University of Tasmania, AUSTRALIA

## Abstract

**Introduction:**

Obesity and overweight strongly contribute to increasing cardiovascular morbidity and mortality, and are becoming a worldwide health issue. The prevalence of obesity has increased dramatically in Latin America. Child obesity is a major issue. Fast food is strongly suspected of contributing to this epidemic of obesity, although there is a lack of evidence.

**Methods:**

We studied the correlation between the number of McDonald restaurants and overweight and obesity prevalence by region stratified by gender and age. Data on prevalences were obtained within national studies conducted by the Brazilian Ministry of Health. Three age sub-groups were analyzed: 5 to 9-year-olds, 10 to 19-year-olds and over 19-year-olds.

**Results:**

There was a very strong positive correlation between overweight rates and the number of McDonald restaurants for both males and females between 5 and 9 years old (R^2^ respectively = 0.92 and 0.84) and a strong positive correlation for females between 10 and 19 years old (R^2^ = 0.68).

There was a very strong positive correlation between obesity rates and the number of McDonald restaurants for males between 5 and 9 years old (R^2^ = 0.95). This positive correlation was strong for both males and females between 10 and 19 years old (R^2^ respectively = 0.77 and 0.63).

Other correlations were not significant.

**Conclusion:**

A strong correlation between the prevalence of overweight and obesity and the number of McDonald restaurants was found for Brazilian children and was most important within the group of youngest children. These results should be taken into consideration by education and prevention campaigns.

## Introduction

Obesity and overweight strongly contribute to increasing cardiovascular morbidity and mortality, and are becoming a worldwide health issue.[1] Both the number of countries and the proportion of population affected are continuously increasing. In 2016, over 39% of adults were overweight and over 13% had obesity according to the World Health Organization (WHO).[2] However, countries and continents aren’t affected equally. Most affected are western countries. In the United States, one third of the population is affected by obesity. In contrast, in Africa, 13% of adults are overweight and 8% have obesity.

The prevalence of obesity has increased dramatically in Latin America. In Brazil, obesity rates increased from 11.6% in 2006 to 17.4% in 2012.[3] Two populations are particularly affected, women and children.[4,5] Obesity in children is a major issue and is well known to be a predictive factor of adult obesity.[6] It is responsible for specific diseases such as type 2 diabetes mellitus, hypertension, nonalcoholic fatty liver disease, obstructive sleep apnea, and dyslipidemia.[6]

Modern ways of life and especially changes in our eating habits, as well as sedentary lifestyles, have largely contributed to increasing obesity. Fast food is strongly suspected of contributing to this epidemic of obesity. We recently reported a strong correlation between a country’s number of McDonald restaurants and overall obesity rates.[7] Number of McDonald restaurants can be considered a strong indicator of change in local population lifestyles. Children are the main target for these kinds of restaurants. There is no specific evidence of the relation between fast food and childhood obesity.

Due to the crucial issue of child obesity in Brazil, we decided to study the correlation between fast food restaurant presence and obesity–overweight rates in the Brazilian population.

## Method

Brazil is geopolitically divided into five macro-regions, each of which has its own economical and sociocultural pattern. These divisions were used as the unit of comparison in our study. The “Pesquisa de Orçamentos Familiares” is a study of nutritional and economical facts of the population that was performed by the Brazilian Ministry of Health (https://biblioteca.ibge.gov.br/visualizacao/livros/liv50063.pdf). Their results were presented by age groups of 5 years. The last version (2008–2009) of this document was used as a reference for obesity rates in each region. Obesity and overweight were defined according to World Health Organization references (http://www.who.int/growthref/who2007_bmi_for_age/en/). Obesity was defined as more than two standard deviations over expected weight for patient’s age and overweight as more than one standard deviation over expected weight for patient’s age. These thresholds are equivalent to respectively BMI > 30 and 25kg/m2 in adult.

Results were stratified by gender and age groups. We studied three age sub-groups, 5 to 9-year-olds, 10 to 19-year-olds and over 19-year-olds.

The exhaustive list of all McDonald restaurants in Brazil was obtained from the AggData website (https://www.aggdata.com/). The Brazilian Demographic Census provided the population per region. With these two data, we calculated the ratio of “McDonald restaurants per million inhabitants per region”.

We then studied the correlation between McDonald restaurant presence and population affected by obesity or overweight in each region. For each analysis, we calculated the determination coefficient (i.e. R squared). It was considered very strong when superior to 0.8 and strong when superior to 0.6 [8] In other cases correlation was considered not significant. We used R software (v3.1.0).

## Results

Total population was 199,492,433 inhabitants, ranging from 14,702,592 to 84,046,162 depending on the region. Population of children aged 5 to 9 years old were 16,009,509 (8%), ranging from 7% to 11% depending on the region. The total number of McDonald restaurants in Brazil was 786, ranging from 14 to 528 depending on the region. The number of McDonald restaurants per million inhabitants was 3.9, ranging from 0.8 to 6.3 depending on the region. Details are in Table 1. Overweight and obesity rates per million inhabitants were at their maximum in the population of over 19 year-olds in both males and females.

**Table 1:** Characteristics of the studied populations and the presence of McDonald restaurants in the five Brazilian regions.

	Region 1 Norte	Region 2 Nordeste	Region 3 Centro-Oeste	Region 4 Sul	Region 5 Sudeste	Brazil Total
Population	16,597,770	55,518,744	14,702,592	28,647,113	84,046,162	199,492,433
Age: 5–9 years	1,766,485	5,015,010	1,197,681	2,024,212	6,006121	16,009,509
Age: 10–19 years	3,514,508	10,246,989	2,588,315	4,551,930	13,279,118	34,180,860
Age > 19 years	10,267,116	36,311,611	10,291,667	20,553,108	60,216,558	137,640,060
McDonald restaurants	14	84	49	111	528	786

### • Overweight

**Fig 1:**
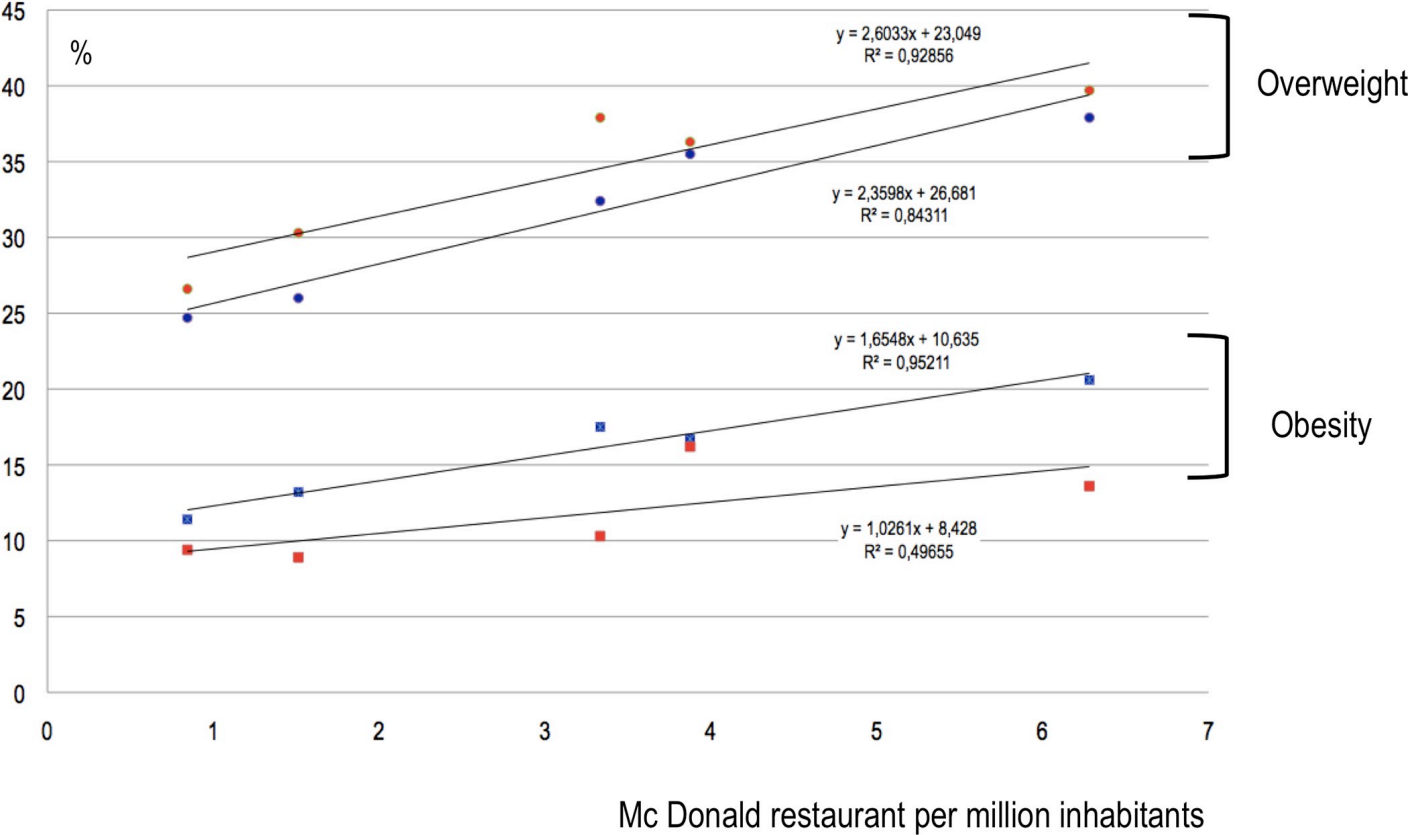
Obesity and overweight prevalence (%) in 5-9-year-old females (respectively red squares and circles) and males (blue squares and circles) in each region according to the number of McDonald restaurants per million-inhabitants.

**Fig 2:**
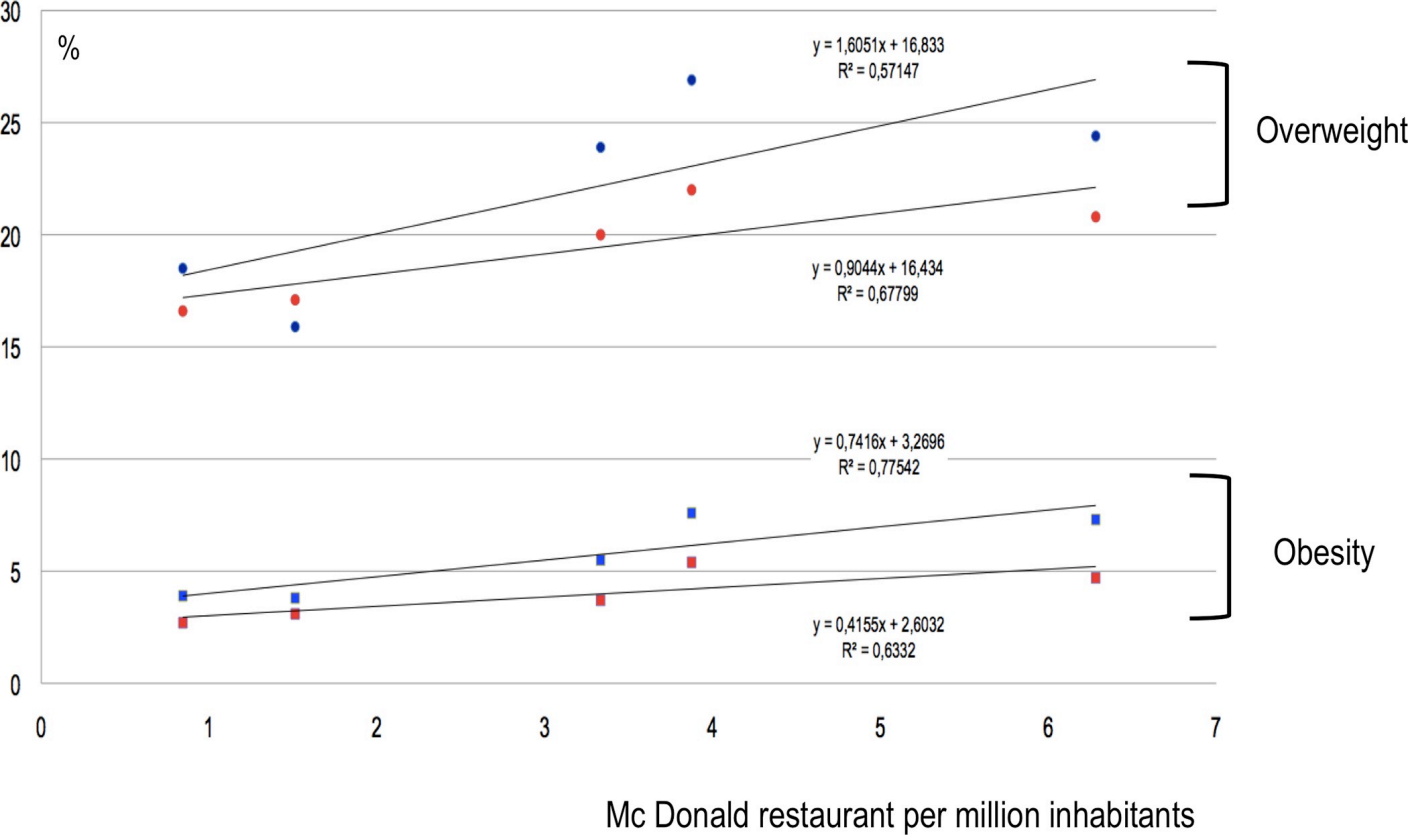
Obesity and overweight prevalence (%) in 10-19-year-old females (respectively red squares and circles) and males (blue squares and circles) in each region according to the number of McDonald restaurants per million-inhabitants.

**Fig 3:**
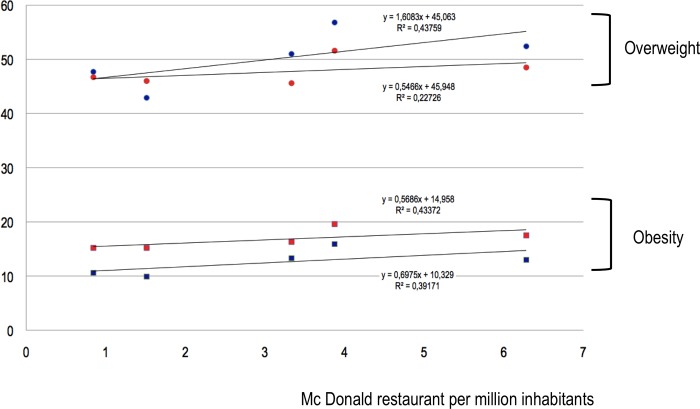
Obesity and overweight prevalence (%) in > 19-year-old females (respectively red squares and circles) and males (blue squares and circles) in each region according to the number of McDonald restaurants per million-inhabitants.

The correlation between overweight and the number of McDonald restaurants was very strong for both males and females between 5 and 9 years old (R^2^ respectively = 0.92 and 0.84) (Fig 1). The correlation was strong for females between 10 and 19 years old (R^2^ = 0.68) (Fig 2). Other correlations were considered not significant (Fig 3).

### • Obesity

The correlation between obesity rates and the number of McDonald restaurants was very strong for males between 5 and 9 years old (R^2^ = 0.95) (Fig 1). The correlation was strong for both males and females between 10 and 19 years old (R^2^ respectively = 0.77 and 0.63) (Fig 2). Other correlations were considered not significant (Figs 1–3).

## Discussion

A strong or very strong correlation between the prevalence of Brazilian children affected by overweight or obesity and the number of McDonald restaurants was found in many subgroups of gender and age class. The highest correlation—i.e. a very strong correlation—was observed for the youngest children. We also noted that this correlation seemed stronger for males than for females. These results should be taken into consideration by education and prevention campaigns.

The correlation between prevalence of overweight or obesity and the number of McDonald restaurants per region in Brazil that we found was weaker than the correlation that we previously reported on a global scale.[7] In a worldwide analysis we found a linear, very strong (R^2^ = 0,95) correlation between overweight and number of McDonald restaurants. In this last study, the conditions for highlighting such a correlation were favorable as the analysis covered 75% of the world population and especially because, in this large sample, the prevalence of people affected by overweight in the population ranged from 2% (Viet-Nam) to 32% (United States) and the number of McDonald's restaurants per million inhabitants from 0 (Viet-Nam) to 45 (United States). In contrast, in Brazil, the prevalence of population affected by obesity ranged from 3% (females 10 to 19 years in the Norte) to 21% (males 5 to 9 years in the Sudeste) and the number of McDonald restaurants per million inhabitants ranged from 0.8 to 6.3. Although the correlation we found here is less statistically strong, the trends all go in the same direction, whichever age category or gender is considered. The appearance of these curves (Figs 1–3) leaves little room for doubt as to the existence, in Brazil, of a correlation between the number of McDonald restaurants per million inhabitants and the prevalence of overweight and obesity. The proximity of fast food restaurants seems to be a factor associated with overweight and obesity, in adults, as well as in children.[7,9]

The strongest correlations were for boys from 5 to 9 years old (R^2^ respectively = 0.93 and 0.95) and for overweight, for girls from 5 to 9 years old (R^2^ = 0.84). This observation is crucial for several reasons. It is clear that children who are affected by overweight and especially obesity have an increased risk of being affected by obesity in adulthood. In fact, the risk for a child with obesity of having obesity in adulthood is multiplied, depending on the gender, by 5 to 9.[10] More generally, childhood obesity is associated with increased medical complications—especially cardiovascular—in adulthood.[11] Childhood diet determines the tastes and eating habits of the teenager and the adult. This ultimately contributes, directly or indirectly, to developing obesity or an ability to control one’s weight. Finally, because children, and more specifically young children, are a preferred commercial target for McDonald's, the correlation was lower for older children and was missing in children over 19 years. It should be noted that this last sub-group was not exposed to McDonald restaurants in the 1990s.[12]

This particularly sensitive subject led certain American jurisdictions to only authorize offering a gift with a meal on the condition that the meal conform to certain nutritional values.[13] Interestingly, a recent study revealed that children will chose the healthiest menu if it comes with a gift.[13] Various educational tools such as the simple question ‘‘what would Batman eat?” have been suggested.[14]

Obesity is already identified as a major health issue in Brazil.[15] In Brazil, contrary to what is observed in Western countries like the United States, the female population is predominantly affected by obesity. This may change. Indeed, the prevalence of obesity in males was significantly higher than that of obesity in females in all regions before the age of 19. The greater the gap between male and female obesity rates, the greater the general obesity rates. Identifying subgroups of population (by age, gender, geographical area) that are most at risk of having overweight or obesity is crucial to optimizing prevention campaigns. The results of this study contribute to this. Taking charge of the '' epidemic '' of overweight and obesity is a very real public health challenge worldwide.[16] Moreover, the interaction between obesity risk factors in children is complex, as shown by a recent Brazilian study.[17]

Such results do not mean that McDonald restaurants are the first responsible for obesity. The establishment and attendance of McDonald restaurants are in fact indicators, among others, of a change in lifestyles, and diet is only one of its components. Thus, analysis carried out with other criteria such as the consumption of sodas, time spent watching television or playing video games or, conversely, the practice of sport, would certainly obtain fairly similar results.[18,19] Many behavioral factors interfere with diet and weight.[20] In Brazil, the amount of McDonald restaurants has grown exponentially over the past 25 years.[12] The number of televisions and refrigerators in households followed the same trend.[12] In our study disparities were observed between rural and urban areas.[12] It is important to note however, that Brazil is already pointing the finger at McDonald’s…[21]

### Limitations of the study

Our results may suffer some criticism. Our analysis focused exclusively on the McDonald's chain. Other fast-food chains could also play a role in the results of this study, as well as other types of food ignored here. However the latter is unlikely, as the correlation between fast food consumption and childhood obesity has been demonstrated worldwide.[22] Causes of overweight and obesity remain multiple and complex.[23] The confounding factors could not be taken into account in this study. Moreover, the validity of our results in other countries cannot be affirmed, although once more, the universality of the correlation has already been demonstrated.[7] Because this correlation was established in a single country study that only analyzed five regions and had a limited range in number of restaurants and in prevalence of obesity and overweight, it is more likely to support a strong relationship. Analysis in other countries will have to confirm this correlation. The reference values used for overweight and obesity are from 2008–09. The impact of the dated data on our interpretation could only be penalizing as the prevalence of overweight and obesity increases, similarly to an epidemic.

## Conclusion

A strong correlation between overweight and obesity rates and the number of McDonald restaurants has been found in the population of Brazilian children. This correlation was at its maximum for the youngest children and for males. Fast-food consumption must be maintained as a main target in the battle against childhood obesity.

## Competing interest statement

All authors have completed the Unified Competing Interest form (available on request from the corresponding author) and declare: no support from any organisation for the submitted work; no financial relationships with any organisations that might have an interest in the submitted work in the previous three years, no other relationships or activities that could appear to have influenced the submitted work.
